# TeaAS: a comprehensive database for alternative splicing in tea plants (*Camellia sinensis*)

**DOI:** 10.1186/s12870-021-03065-8

**Published:** 2021-06-21

**Authors:** Xiaozeng Mi, Yi Yue, Mengsha Tang, Yanlin An, Hui Xie, Dahe Qiao, Zhiyu Ma, Shengrui Liu, Chaoling Wei

**Affiliations:** 1 State Key Laboratory of Tea Plant Biology and Utilization, Anhui Agricultural University, West 130 Changjiang Road, Hefei, Anhui 230036 People’s Republic of China; 2 School of Information and Computer, Anhui Agricultural University, West 130 Changjiang Road, Hefei, Anhui 230036 People’s Republic of China

**Keywords:** Alternative splicing, Genome and transcriptome, Transcripts, Isoforms, Tea plant

## Abstract

Alternative splicing (AS) increases the diversity of transcripts and proteins through the selection of different splice sites and plays an important role in the growth, development and stress tolerance of plants. With the release of the reference genome of the tea plant (*Camellia sinensis*) and the development of transcriptome sequencing, researchers have reported the existence of AS in tea plants. However, there is a lack of a platform, centered on different RNA-seq datasets, that provides comprehensive information on AS.

To facilitate access to information on AS and reveal the molecular function of AS in tea plants, we established the first comprehensive AS database for tea plants (TeaAS, http://www.teaas.cn/index.php). In this study, 3.96 Tb reads from 66 different RNA-seq datasets were collected to identify AS events. TeaAS supports four methods of retrieval of AS information based on gene ID, gene name, annotation (non-redundant/Kyoto encyclopedia of genes and genomes/gene ontology annotation or chromosomal location) and RNA-seq data. It integrates data pertaining to genome annotation, type of AS event, transcript sequence, and isoforms expression levels from 66 RNA-seq datasets. The AS events resulting from different environmental conditions and that occurring in varied tissue types, and the expression levels of specific transcripts can be clearly identified through this online database. Moreover, it also provides two useful tools, Basic Local Alignment Search Tool and Generic Genome Browser, for sequence alignment and visualization of gene structure.

The features of the TeaAS database make it a comprehensive AS bioinformatics platform for researchers, as well as a reference for studying AS events in woody crops. It could also be helpful for revealing the novel biological functions of AS in gene regulation in tea plants.

**Supplementary Information:**

The online version contains supplementary material available at 10.1186/s12870-021-03065-8.

## Background

Alternative splicing (AS) refers to pre-mRNA processing events that produce multiple mRNAs from the same gene through variable selection of splice sites. It is an important mode of regulating gene expression and increasing protein diversity [1]. AS can be grouped into four main types: intron retention (IR), alternative 3' splice sites (A3SS), alternative 5' splice sites (A5SS), and exon skipping (ES). AS has been found to be involved in growth and development of cell types, metabolism, and regulates the response to biological and abiotic stress in many plant species [2–4]. For example, the FLOWERING LOCUS M (*FLM*) is subject to temperature-dependent AS that regulates flowering in Arabidopsis [5]. In addition, the gene involved in the AS of an MYB transcription factor affects anthocyanin biosynthesis in tomato fruits, and mutations in the splice sites cause a complete loss of function in the wild-type protein [6]. Moreover, *LlHSFA3B-III*, an AS isoform of *LlHSFA3B* (heat stress transcription factors), was also found to regulate abiotic heat stress in lily plants [7]. In tea plants, an AS-associated analysis of different tissues showed that it could regulate the flavonoid pathway through certain transcription factors and functional genes [8]. In response to cold stress, plants undergo AS to regulate expression of genes involved in sugar metabolism pathways and those coding for antioxidant enzymes [9]. Furthermore, AS isoforms of the *CsLOX* gene were generated by tea geometrid feeding and *Glomerella cingulata* infection. Thus, AS plays an important role in plant growth, development and response to stress. AS databases have been established in many other crops, such as cucumber [10]. In addition, a comprehensive AS database of plant communities was established, including that of cotton [11], tomato [12], fruit plants [13], Brachypodium distachyon [14] and other crops. The establishment of these databases enriches the knowledge on gene functions and facilitates access to AS-related information; however, till date, there is no comprehensive database of AS in tea plants (*Camellia sinensis*).

The tea plant is an important perennial evergreen woody crop of significant economic and cultural importance [15]. Tea leaves are used for making tea beverages, which is one of the three most popular nonalcoholic beverages consumed worldwide. It not only contains polyphenols, catechins, caffeine, and other flavorful compositions, but it also provides health-promoting benefits [16, 17]. Several databases have been established to facilitate the study of tea plants [18–20], however, these do not include the information associated with AS.

With the release of the reference genome of the tea plant [21–23], researchers have made great progress in the understanding of functional genes and their regulatory mechanisms, which also provides a basis for the identification of AS. Transcriptome sequencing has become an important way to study the biological functions of genes. Recently, transcriptomes in response to different environmental conditions have been analyzed in many plant species [24, 25]. In tea plants, transcriptome sequencing has been used to understand the expression of genes responsive to low temperature, salinity, drought, and heat stress [9, 26, 27], and also to reveal the mechanisms of self-incompatibility, secondary metabolism, and development [28–30]. These studies have enriched the understanding of the biological function of genes in tea plants, but they only focused on a single transcript derived from each gene, and did not investigate the function of multiple transcripts arising from AS events. Therefore, the role of AS in these biological processes in tea plants is unclear. Mapping the RNA sequence (obtained by RNA-seq) to the reference genome and assembling the genomes are important steps to identify AS events, which promote the understanding of the biological function of AS. Thus, the objective of this study was to establish a comprehensive AS database of tea plants, the TeaAS, to provide researchers with a public and freely available AS database.

## Construction and content

### Data sources

The genome of the tea plant (*Camellia sinensis* var. *sinensis* cv. suchazao) was used as the reference genome [21]. The current release of the tea plant genome and genome annotations were collected from the TPIA platform (http://tpia.teaplant.org/). Sixty-six RNA-seq datasets associated with different environmental conditions or tissue types were downloaded from the SRA database (https://www.ncbi.nlm.nih.gov/sra/). Details of all RNA-seq data are provided in Additional file 1: Table S1.

### Data processing

The SRA Toolkit was used to download RNA-seq data from the NCBI Sequence Read Archive (SRA) database. All raw sequencing data were filtered through the Trimmomatic (v0.36) software with the same standard [31]. The adapter sequence was removed by the ILLUMINACLIP parameter with a minimum adapter length of eight. In addition, reads with a continuous 5 bp base average quality below 20 and a length under 36 bp after filtering were filtered out. The remaining paired reads were used for subsequent analyses.

The filtered RNA-seq reads were mapped to the reference genome using Hisat2 (v2.1.0) with default parameters. The generated sequence alignment map (SAM) files were converted to BAM files by Samtools software. Then transcripts were assembled using StringTie software (v.1.3.5) with default settings [32]. Finally, the gene transfer format (GTF) files from each sample in the same transcriptome were merged using the StringTie with “–merge” parameter.

### Identification of AS events

The online tool AStalavista (http://astalavista.sammeth.net/) was employed to identify the AS events from different transcriptome datasets [33]. Briefly, the merged GTF files were submitted to the website by selecting other organisms, and the other options used default values. Four major types of AS events, including IR, A3SS, A5SS, ES, and multiple splicing events occurring on a single transcript were extracted from the output files and counted, respectively. The reads number of splice sites and differential expression of the AS genes were analyzed by rMATS (v.4.1.1, rnaseq-mats.sourceforge.net/) software [34].

Transcripts per million reads (TPM) were used to quantify the transcript expression levels. The TPM from each sample was calculated using StringTie. Then, the gene ID, transcript ID, and their TPM were extracted to form a dataset of all the transcript expression levels. To avoid the effect of very weakly expressed transcripts on the accuracy of the results, we filtered out transcripts with TPM less than one in all the samples. A gene is considered to have undergone AS only if it has at least two mRNAs are produced from the precursor-mRNA and the TPM of these transcripts is greater than one in at least one sample.

### Functional annotation of transcripts

All amino acid and nucleotide sequences of the full-length transcripts and AS isoforms were extracted using GTF files generated by Stringtie. NR, GO and KEGG annotation databases were used to compare all transcripts to the annotations of the reference genome. The coding region and amino acid sequence of the newly generated transcripts were predicted using the TransDecoder software (https://github.com/TransDecoder/TransDecoder, v5.5.0). Only one coding sequence (CDS) per transcript was the output using the “–single_best_only” parameter and homology search against the UniProt database. To prevent the generation of nonsense codons or short peptides, we used any amino acids greater than or equal to 100 aa for further analysis. The domains information of all amino acids was identified using the Pfamscan software (ftp://ftp.ebi.ac.uk/pub/databases/Pfam/Tools/).

## Utility

**Fig. 1:**
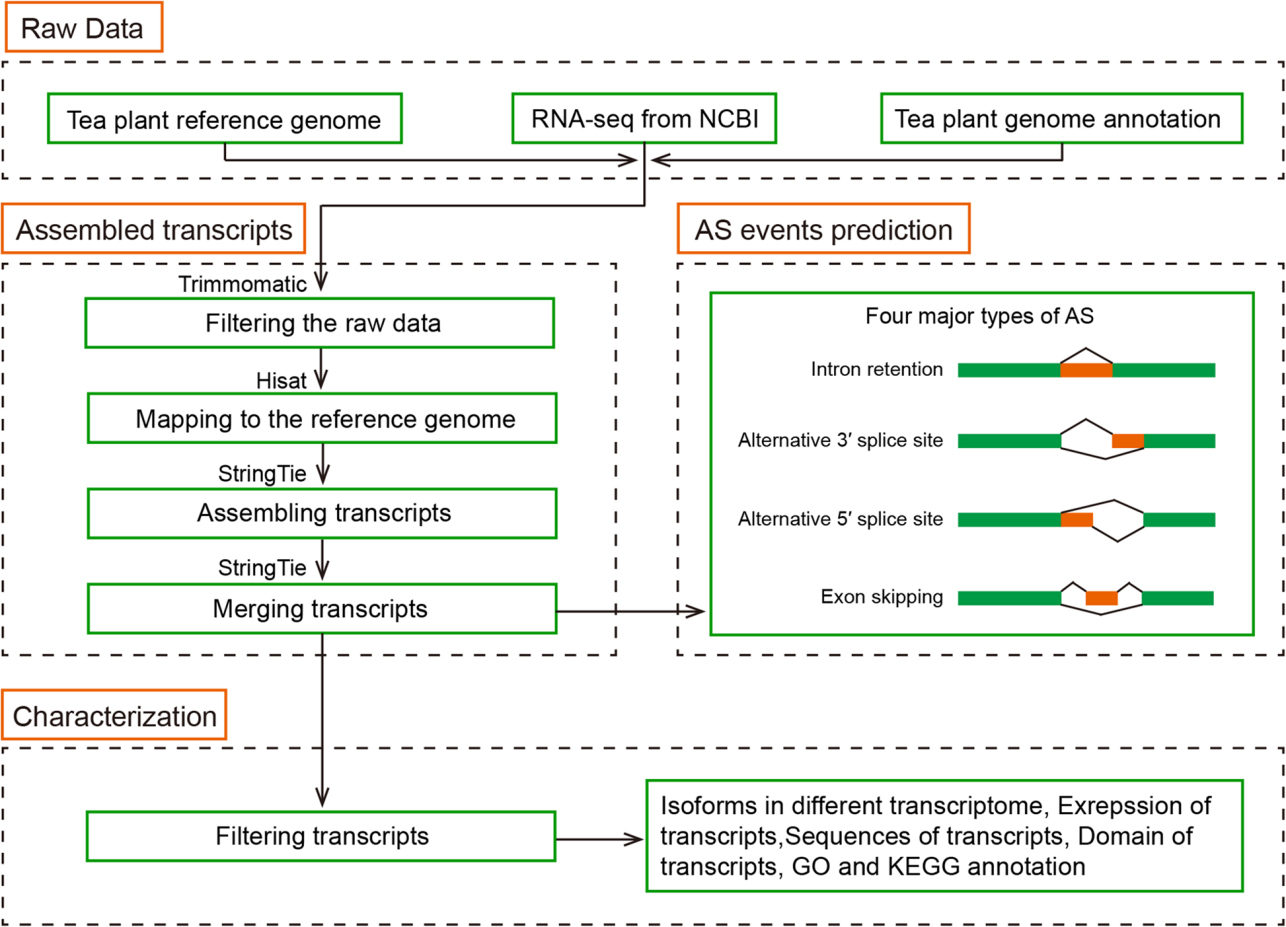
Workflow of the construction of the TeaAS database

A multistep process was used to construct the TeaAS database (Fig. 1). The TeaAS web interface offers four main functional sections, including Home (search), Summary, Tools, and Download, to provide information on the AS. In addition, the links section provides users with links to the genome and other databases of tea species, Users can provide feedbacks through the “Contacting Us” section.

### General functions

**Fig. 2:**
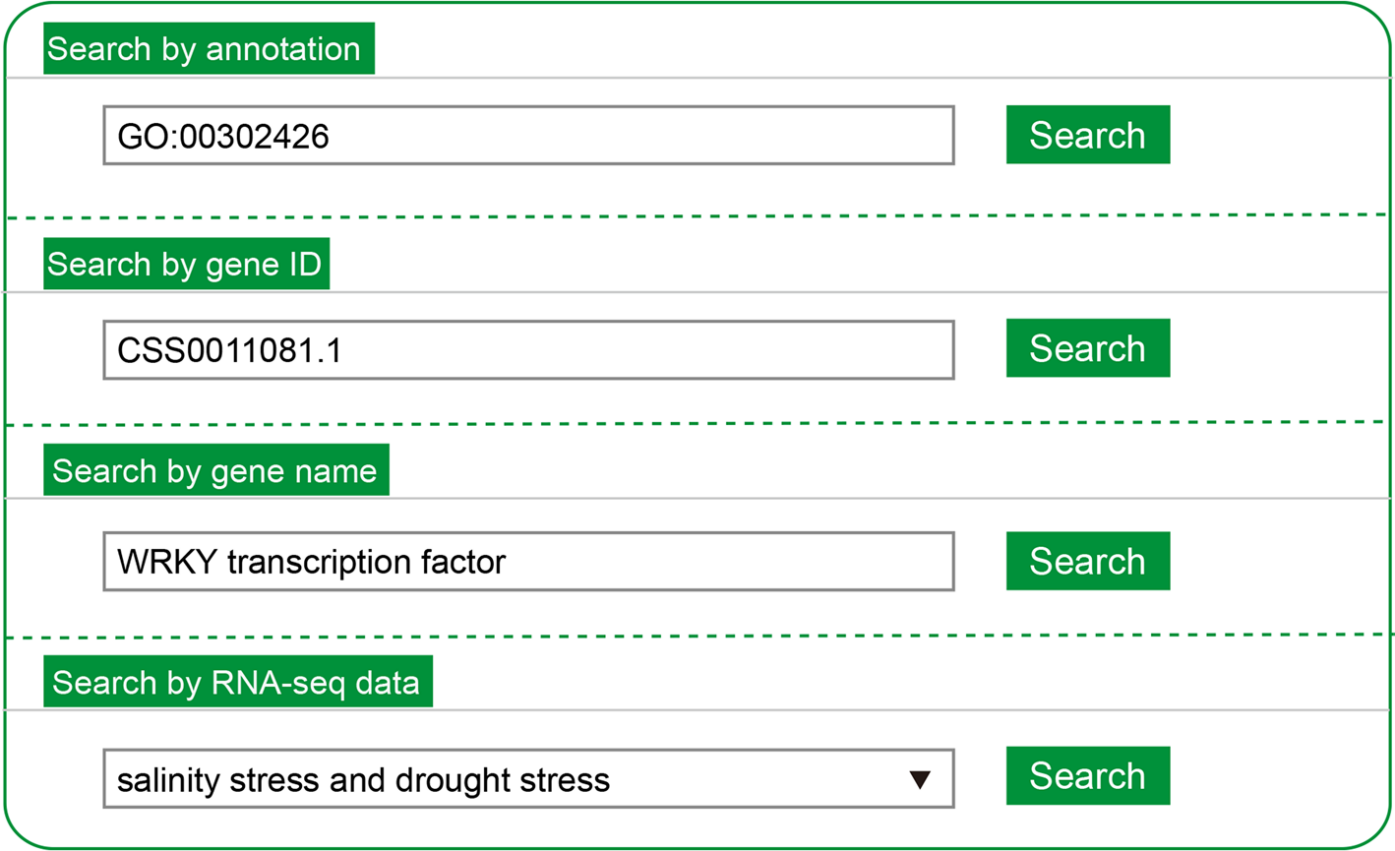
Search tools of TeaAS

**Fig. 3:**
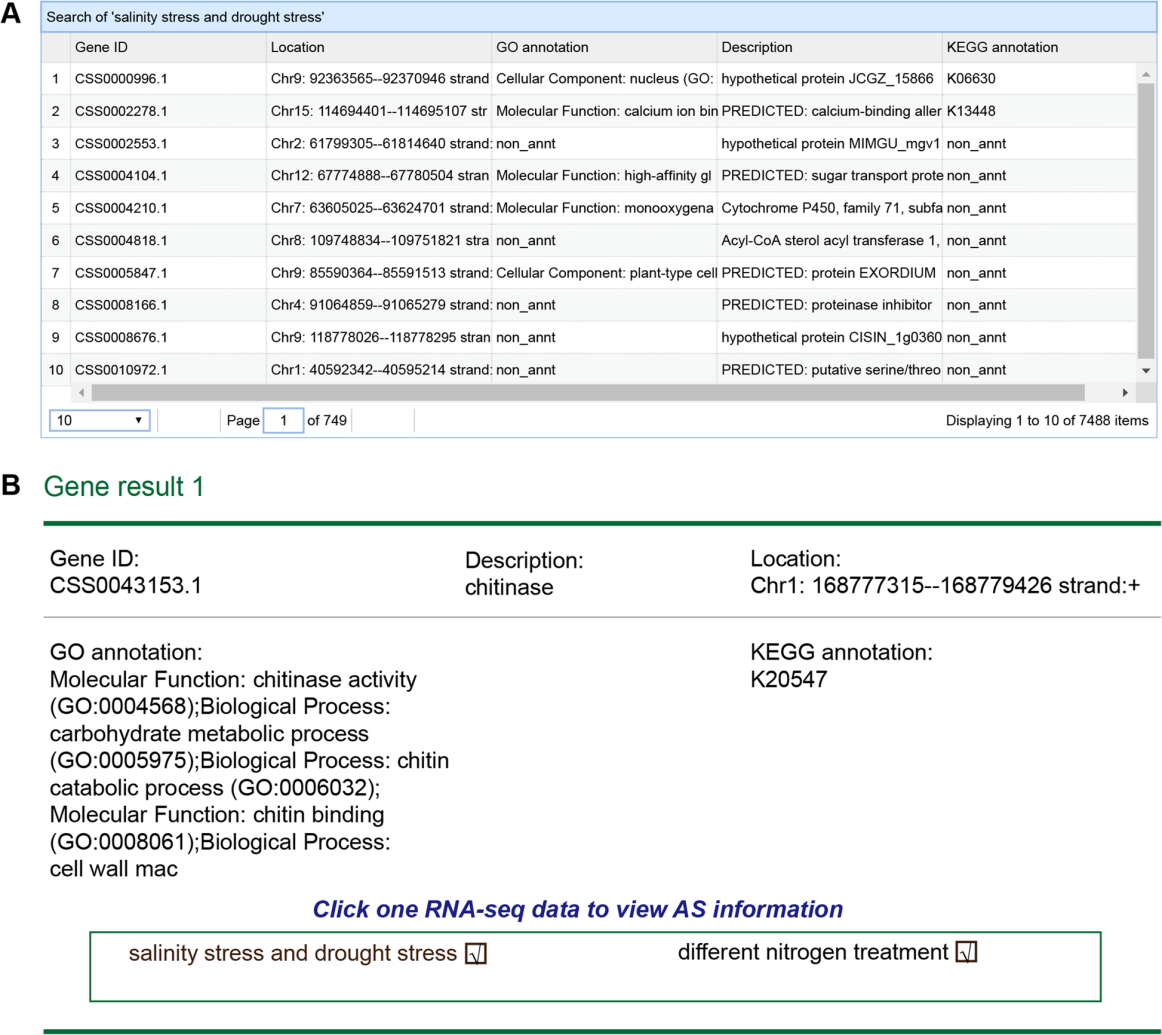
Search results by gene ID, gene name and RNA-seq datasets. **a** The list of genetic information based on gene annotation and RNA-seq datasets; and** b** results page upon searching by the gene ID and gene name

The home page contains the search interface (Fig. 2). The users can search the database using the gene ID (e.g., CSS0013065.1), gene name (e.g., bZIP transcription factor), gene annotation [non-redundant (NR), Kyoto Encyclopedia of Genes and Genomes (KEGG), gene ontology (GO) annotation, and chromosomal location], and RNA-seq datasets (e.g., drought stress). Search results are represented on the results page in two ways. First, a list of genes and associated information would be presented if the user conducted the search for all the above-mentioned annotations and for varying environmental conditions (Fig. 3a). Each gene in the list can be double-clicked to determine the environmental condition upon which an AS event has occurred. The transcript and protein products of the AS event can be visualized after a gene and treatment are selected. Second, the environmental conditions and genetic information are rendered when the user searches with gene IDs and gene families (Fig. 3b). Subsequently, the details of the AS event can be browsed by clicking on one environmental condition.

### Database overview

Data and statistical information can be found on the summary page (Additional file 2: Figure S1). This page provides: (I) the transcriptome information, which includes an introduction to the transcriptome, the NCBI accession number, information for each treatment sample, and corresponding publications; (II) statistics of the four major types of AS events and the number of AS genes (Additional file 3: Table S2); (III) the percentage of AS genes among all annotated genes; (IV) the distribution of AS genes on 15 chromosomes; (V) annotation information of all AS genes; and (VI) percent spliced in (PSI) between treatments and the number of reads at splice sites (Additional file 4: Table S3).

The results showed that a total of 131,924 AS events were identified in 11,320 genes in the transcriptome of PRJNA576575, whereas only 9,152 AS events were identified in a self-incompatibility transcriptome (PRJNA355226). This may be due to the differences in treatment conditions of the tea plants and the size of the sequencing data. A total of 28,648 genes underwent AS, which accounts for 56.7% of the total number of annotated genes (50,525). Moreover, only 0.56% of the genes that underwent AS were common in all the transcriptome datasets.

### Other functions

TeaAS mainly provides users with two effective tools, Basic Local Alignment Search Tool (BLAST) [35] and Generic Genome Browser (GBrowse). BLAST can be used to find homologous sequences in the tea plant. Users can compare the query sequences with any of the assembled transcripts from the 66 transcriptome datasets. The results could then be analyzed for the occurrence of AS events. GBrowse was used to visualize the gene structure of tea plants, including the transcripts from 66 transcriptome datasets.

The download page allows users to download all the data for free. The assembled GTF files, AS event identification files, and all transcription expression files from 66 transcriptome datasets can be downloaded from this page. Moreover, the results of the search can also be downloaded directly. In addition, TeaAS provides a link to the tea plant genome website and to the other tea plant databases on the link page.

### Usage cases

Case study 1, To search for AS events in a gene: The users can directly use the gene name and gene ID to search whether the gene is capable of undergoing AS. At the same time, users can also submit a sequence for confirming the gene ID by the BLAST tool. For example, a search with the ID CSS0005154.1 yields the result that the gene encodes a NAC transcription factor and undergoes AS events under multiple treatment conditions, such as salt stress, exposure to sucrose, and cold acclimation. In addition, users can identify genes that undergo AS a specific treatment. For instance, many disease-resistant genes and transcription factors undergo AS under conditions of gray blight disease.

**Fig. 4:**
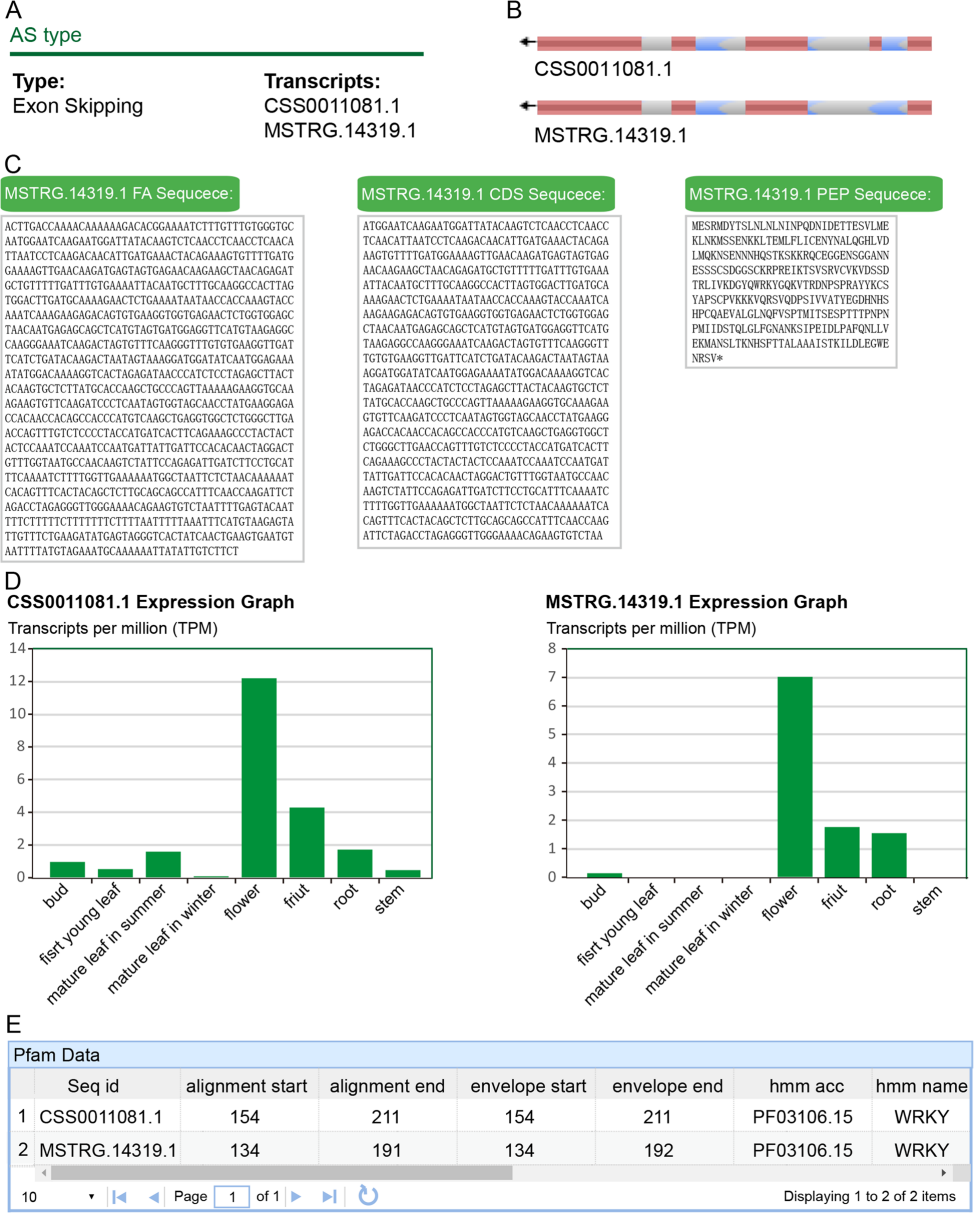
Alternative splicing information of TeaAS. **a** AS type and transcripts ID; **b** gene structure of full-length transcripts and AS isoforms; **c** nucleotide and amino acid sequences of transcripts; **d** expression levels of transcripts in each sample, and **e** domain information of transcripts

Case study 2, To obtain details associated with AS genes: For example, retrieval based on the gene ID CSS0011081.1 yields the WRKY gene, which is located on chromosome 14, encodes a transcription factor,and has no GO and KEGG annotations. Furthermore, the conditions under which AS events have occurred can also be identified, such as anthracnose diseases, in different tissue types, and upon shade treatments. After clicking on “different tissues 2,” we determined that this gene undergoes AS and yields two transcripts, CSS0011081.1 and MSTRG.14319.1 (Fig. 4a). The structure of these two transcripts is shown in Fig. 4b, where the second exon of the AS isoform is missing. The RNA sequence, coding sequence (CDS), and amino acid sequences of the transcripts can be viewed directly from the sequence options (Fig. 4c). The two transcripts were found to have the highest expression levels in the flowers through the data of expression levels (Fig. 4d). Although a 30 bp nucleic acid sequence was lacking in the AS isoform, this sequence was not present in the WRKY domain (Fig. 4e).

## Discussion

AS is a gene regulatory mechanism that has been shown to play an important role in plant growth and development and in responses to biotic and abiotic stresses [1, 2, 36]. In tea plants, AS is involved in gene expression associated with metabolism [37], response to low temperature [9], and drought stress [38]. However, there is a lack of data available for researchers regarding AS in tea plants. In this study, we have established, for the first time, a comprehensive AS database for tea plants. The datasets included in our database can be used as a reference for AS, and the abundant transcriptome data can provide basic information on gene function.

AS databases have been reported in other plants. For example, an AS database for cucumbers, named CuAS, provides detailed information on AS events and transcripts [10]. This database provides information on the AS isoform sequences, domains, and expression levels. In addition, a large number of tissue-specific AS events have been identified in CuAS. However, sequencing data from only 17 different tissues of two cucumber species were included in this database, which is far from what is sufficient to fully understand the AS in cucumber plants. Another AS database was established in tomatoes [12], wherein 300,665 expressed sequence tag sequences and 27 transcriptome datasets were used to identify AS. Compared to the cucumber database, the tomato database represented a variety of tissue types, however, it only provided a search page with limited AS information. To enrich the content of the database established in this study, we collected 66 transcriptome datasets obtained upon exposure to different environmental conditions for a total of 3.96 Tb sequencing reads, making the identification of AS in tea plants accurate and comprehensive. Additionally, more information about full-length transcripts and AS isoforms is presented on our website, and useful tools for analysis are provided. Thus, TeaAS can be used as a reference for the AS in tea plants. With the publication of a high-quality tea plant genome sequence, the Tea Plant Information Archive (TPIA) was established [18], which contains information on the tea plant genome, transcriptome, and metabolome. It provides a powerful tool for analyzing genomic data and elucidating biological mechanisms of growth and development in tea plants. The database of simple sequence repeat (SSR) markers [19] and co-expression networks [20] have been established in tea plants, providing a basis for the study of functional genes and breeding in tea plants. However, a database related to AS has not been reported yet. The establishment of TeaAS would provide an important tool for studying gene regulation in relation to AS in tea plants.

TeaAS provides a comprehensive information on AS by mapping RNA-seq data to the reference genome. In recent years, with the development of sequencing technology, numerous transcriptome datasets have been sequenced. For instance, transcriptome sequencing has revealed that several genes involved in sugar metabolism, proline metabolism, induction of CBF (C-repeat binding factor) expression, and the cold-responsive (ICE—CBF – COR) pathway play key roles in cold acclimation in tea plants [39]. Using the same data for AS analysis, researchers found that AS could also affect the sugar metabolism pathway. Furthermore, AS may respond to low temperatures via oxidoreductase and transcription factors (e.g. bHLH, WRKY) [9]. In addition, there have been several reports identifying genes related to anthocyanin synthesis in tea plants [40, 41]. In fact, the synthesis of anthocyanin was not only regulated by the full-length transcripts, but also by AS isoforms of genes such as anthocyanidin reductase (ANR) and anthocyanidin synthase (ANS) [37]. These results indicated that AS plays an important role in regulating metabolism and stress responses of tea plants. To facilitate the acquisition of AS information, TeaAS provides researchers with a simple search interface.

## Conclusions

In summary, TeaAS is a platform that provides comprehensive information on full-length transcripts and their AS isoforms in tea plants. The establishment of TeaAS provides a reference for AS and would be helpful in potential role of AS in gene regulation via comparative analyses in tea plants.

## Supplementary Information

**Additional file 1: Table S1.** Details of 66 RNA-seq dataset.

**Additional file 2: Figure S1.** Statistics of 66 RNA-seq datasets and AS events.

**Additional file 3. Table S2. **Statistics of four major AS events in 66 RNA-seq datasets.

**Additional file 4: Table S3.** Information of differentially expressed AS by rMATS analysis.

## Data Availability

The genomic data of the tea plant (Camellia sinensis var. sinensis cv. suchazao) are available at TPIA platform (http://tpia.teaplant.org/download.html). The RNA-seq datasets supporting the results of this article are available at the SRA database of NCBI (https://www.ncbi.nlm.nih.gov/), and all relevant accession numbers and details are provided in TeaAS (http://www.teaas.cn/static/html/Browse.html).

## Abbreviations

TeaAS: A database for alternative splicing in tea plant
AS: Alternative splicing
IR: Intron retention
A3SS: Alternative 3' splice sites
A5SS: Alternative 5' splice sites
ES: Exon skipping
TPM: Transcripts per million reads
NR: Non-redundant protein
GO: Gene ontology
KEGG: Kyoto Encyclopedia of Genes and Genomes
CDS: Coding sequence
