# Supplementary figures and images for: TeaAS: a comprehensive database for alternative splicing in tea plants (Camellia sinensis)
Source: BMC Plant Biol. 2021 Jun 21;21:280. doi: 10.1186/s12870-021-03065-8 (PMC8215737; doi:10.1186/s12870-021-03065-8)

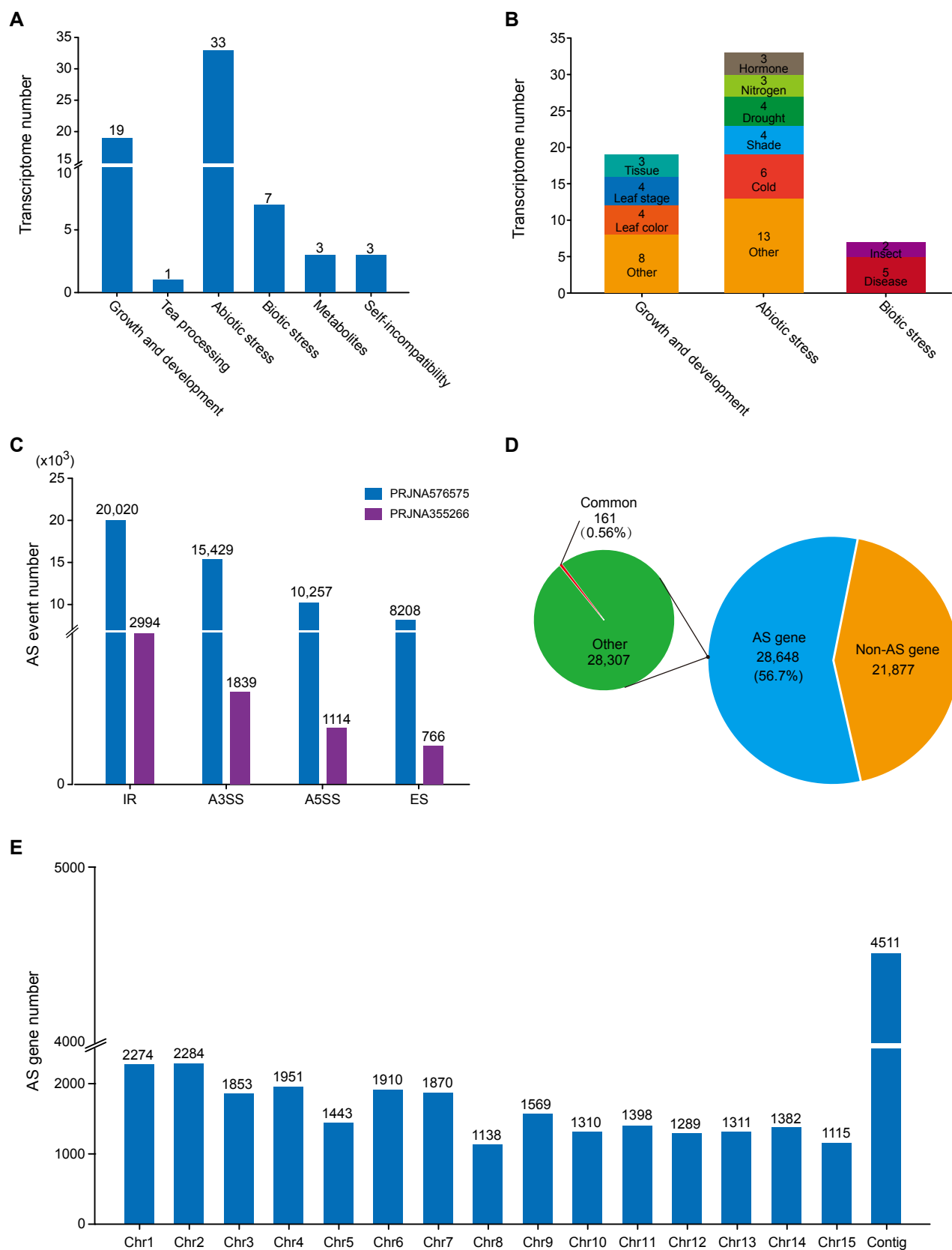

**Fig. S1** Statistics of 66 RNA-seq datasets and AS events.

Supplement: Supplementary file 2 — Additional file 2: Figure S1. Statistics of 66 RNA-seq datasets and AS events. [file 12870_2021_3065_MOESM2_ESM.pdf]
