# Supplementary material for: TeaAS: a comprehensive database for alternative splicing in tea plants (Camellia sinensis)
Source: BMC Plant Biol. 2021 Jun 21;21:280. doi: 10.1186/s12870-021-03065-8 (PMC8215737; doi:10.1186/s12870-021-03065-8)
Supplement: Supplementary file 3 — Additional file 3. Table S2. Statistics of four major AS events in 66 RNA-seq datasets. [file 12870_2021_3065_MOESM3_ESM.docx]

Table S2. Statistics of four major AS events in 66 RNA-seq datasets.

| Accession number | AS events | | | | | | Gene  number |
| --- | --- | --- | --- | --- | --- | --- | --- |
|  | IR | A3SS | A5SS | ES | Other | Total |  |
| PRJEB11522 | 7583 | 6784 | 4001 | 3670 | 10323 | 32361 | 7488 |
| PRJNA178636 | 3840 | 2442 | 1570 | 1192 | 2509 | 11553 | 4235 |
| PRJNA230752 | 12914 | 8870 | 5577 | 4416 | 14960 | 46737 | 10185 |
| PRJNA240661 | 9052 | 6164 | 3850 | 3271 | 8855 | 31192 | 7858 |
| PRJNA257942 | 5578 | 2629 | 1698 | 1083 | 4696 | 15684 | 4794 |
| PRJNA274203 | 10922 | 8177 | 4974 | 3953 | 12462 | 40488 | 9265 |
| PRJNA277458 | 8792 | 5362 | 3322 | 2470 | 10380 | 30326 | 7695 |
| PRJNA288922 | 9718 | 8073 | 5134 | 4117 | 15567 | 42609 | 8733 |
| PRJNA291116 | 10225 | 6860 | 4548 | 3532 | 11967 | 37132 | 7943 |
| PRJNA295355 | 8631 | 5692 | 3617 | 2654 | 8046 | 28640 | 7649 |
| PRJNA297732 | 9587 | 9419 | 5600 | 4568 | 9556 | 38730 | 9614 |
| PRJNA306068 | 3913 | 3182 | 1917 | 1310 | 4538 | 14860 | 4661 |
| PRJNA312027 | 10195 | 7451 | 4685 | 3628 | 15139 | 41098 | 8437 |
| PRJNA318668 | 11543 | 8658 | 5289 | 4437 | 17784 | 47711 | 9620 |
| PRJNA326551 | 9563 | 7579 | 4707 | 3965 | 12747 | 38561 | 8434 |
| PRJNA329179 | 12400 | 8198 | 5118 | 3867 | 14845 | 44428 | 9270 |
| PRJNA340316 | 6119 | 4421 | 2777 | 2394 | 5474 | 21185 | 6373 |
| PRJNA344817 | 5977 | 4164 | 2772 | 2299 | 7114 | 22326 | 6144 |
| PRJNA347510 | 11035 | 10170 | 6198 | 6733 | 28135 | 62271 | 9401 |
| PRJNA348643 | 8193 | 4890 | 3195 | 2220 | 6766 | 25264 | 7134 |
| PRJNA355226 | 2994 | 1839 | 1114 | 766 | 2439 | 9152 | 3555 |
| PRJNA356134 | 7473 | 5882 | 3589 | 2618 | 10776 | 30338 | 7162 |
| PRJNA381680 | 9870 | 5628 | 3708 | 2904 | 8817 | 30927 | 8173 |
| PRJNA387105 | 13564 | 9789 | 6343 | 5543 | 21521 | 56760 | 10072 |
| PRJNA387271 | 12926 | 6449 | 3949 | 3048 | 11215 | 37587 | 9266 |
| PRJNA394625 | 16890 | 11888 | 7950 | 5821 | 39428 | 81977 | 10746 |
| PRJNA396805 | 10083 | 8706 | 5471 | 3441 | 55194 | 82895 | 7819 |
| PRJNA398691 | 8070 | 6750 | 4062 | 3389 | 11760 | 34031 | 7956 |
| PRJNA589096 | 10490 | 6979 | 4509 | 3170 | 14072 | 39220 | 8328 |
| PRJNA383883 | 9306 | 5876 | 3933 | 3254 | 9032 | 31401 | 8159 |
| PRJNA400599 | 9809 | 6825 | 4748 | 3217 | 18725 | 43324 | 8319 |
| PRJNA411886 | 13816 | 8783 | 5834 | 6489 | 18768 | 53690 | 10373 |
| PRJNA413653 | 14909 | 11342 | 7077 | 6476 | 28983 | 68787 | 11041 |
| PRJNA414949 | 3937 | 3597 | 2113 | 1583 | 5637 | 16867 | 4919 |
| PRJNA420937 | 12722 | 7593 | 4831 | 3373 | 17159 | 45678 | 9341 |
| PRJNA422456 | 11298 | 8438 | 5054 | 4288 | 17607 | 46685 | 8922 |
| PRJNA431489 | 7027 | 4579 | 3067 | 2224 | 8818 | 25715 | 6531 |
| PRJNA433795 | 5398 | 3629 | 2254 | 1498 | 4416 | 17195 | 6006 |
| PRJNA439206 | 13738 | 9999 | 6553 | 5073 | 27954 | 63317 | 9877 |
| PRJNA449232 | 7199 | 5724 | 3633 | 2530 | 9790 | 28876 | 7282 |
| PRJNA473228 | 12718 | 9018 | 5754 | 4425 | 16898 | 48813 | 9540 |
| PRJNA473596 | 5648 | 3334 | 2046 | 1428 | 5029 | 17485 | 5357 |
| PRJNA478288 | 8746 | 6174 | 3888 | 3058 | 9927 | 31793 | 7995 |
| PRJNA484299 | 16049 | 11643 | 7995 | 6117 | 54758 | 96562 | 10201 |
| PRJNA492937 | 16893 | 11448 | 7443 | 6457 | 23289 | 65530 | 11022 |
| PRJNA493214 | 8180 | 6774 | 4384 | 3378 | 9688 | 32404 | 7365 |
| PRJNA505084 | 19744 | 15465 | 10156 | 9584 | 47433 | 102382 | 11765 |
| PRJNA512448 | 10593 | 7025 | 4633 | 3334 | 14955 | 40540 | 8394 |
| PRJNA516040 | 12734 | 8571 | 5496 | 3981 | 19203 | 49985 | 9311 |
| PRJNA516116 | 11186 | 7413 | 4521 | 3786 | 16241 | 43147 | 8543 |
| PRJNA517582 | 15488 | 10942 | 6668 | 5032 | 39527 | 77657 | 9862 |
| PRJNA522339 | 17170 | 11219 | 7628 | 5968 | 35141 | 77126 | 11555 |
| PRJNA528172 | 11377 | 8366 | 5206 | 4210 | 15138 | 44297 | 9484 |
| PRJNA528853 | 11223 | 7958 | 5209 | 4248 | 15227 | 43865 | 8988 |
| PRJNA539837 | 9532 | 5715 | 3676 | 2740 | 10363 | 32026 | 7806 |
| PRJNA545401 | 19573 | 12496 | 8570 | 9062 | 34925 | 84626 | 11447 |
| PRJNA553681 | 14239 | 9339 | 6074 | 4534 | 23094 | 57280 | 9396 |
| PRJNA557820 | 6709 | 3463 | 2091 | 1357 | 6058 | 19678 | 5714 |
| PRJNA559220 | 16429 | 10536 | 6779 | 5419 | 28657 | 67820 | 10782 |
| PRJNA563742 | 11406 | 8291 | 5383 | 4108 | 28426 | 57614 | 9194 |
| PRJNA564655 | 18358 | 13272 | 8369 | 6897 | 46589 | 93485 | 10745 |
| PRJNA575691 | 9342 | 7857 | 5131 | 4616 | 17093 | 44039 | 8163 |
| PRJNA576575 | 20020 | 15429 | 10257 | 8208 | 78010 | 131924 | 11320 |
| PRJNA596070 | 15296 | 9662 | 6737 | 5137 | 28697 | 65592 | 9632 |
| PRJNA597433 | 16310 | 13687 | 8349 | 7039 | 27869 | 73254 | 11137 |
| PRJNA602394 | 12917 | 7920 | 5255 | 4066 | 25800 | 55958 | 8816 |
